# Translation and cross-cultural adaptation of the Behavior Change Protocol for educational practices in Diabetes Mellitus*

**DOI:** 10.1590/1518-8345.2908.3164

**Published:** 2019-08-19

**Authors:** Fernanda Azeredo Chaves, Sumaya Giarola Cecilio, Ilka Afonso Reis, Adriana Silvina Pagano, Heloísa de Carvalho Torres

**Affiliations:** 1 Prefeitura de Belo Horizonte, Coordenação de Atenção à Saúde da Mulher, Belo Horizonte, MG, Brasil.; 2 Universidade Federal de Minas Gerais, Departamento de Enfermagem Aplicada, Belo Horizonte, MG, Brasil.; 3 Bolsista da Coordenação de Aperfeiçoamento de Pessoal de Nível Superior (CAPES), Brasil.; 4 Universidade Federal de Minas Gerais, Instituto de Ciências Exatas, Belo Horizonte, MG, Brasil.; 5 Universidade Federal de Minas Gerais, Faculdade de Letras, Belo Horizonte, MG, Brasil.

**Keywords:** Power (Psychology, Health Education, Health Promotion, Validation Studies, Questionnaires, Diabetes Mellitus Type 2, Poder (Psicologia, Educação em Saúde, Promoção da Saúde, Questionários, Estudos de Validação, Diabetes Mellitus Tipo 2, Poder (Psicología, Educación en Salud, Promoción de la Salud, Estudios de Validación, Cuestionarios, Diabetes Mellitus Tipo 2

## Abstract

**Objective:**

to translate and cross-culturally adapt the Behavior Change Protocol for educational practices in Diabetes Mellitus.

**Method:**

methodological study aimed at cross-cultural adaptation, comprising the steps of translation, back-translation, assessment by an expert committee and pre-testing of the instrument on a sample of 30 healthcare service users with type 2 Diabetes Mellitus.

**Results:**

the instrument was assessed based on criteria pertaining semantic, idiomatic, conceptual and cultural equivalence between the original instrument and the translated version, its mean Content Validity Index being 0.85.

**Conclusion:**

results showed content validity indicating the instrument’s successful cross-cultural adaptation to the Brazilian culture for use in educational practices targeting self-care in type 2 DM.

## Abstract (pt)

**Objetivo:**

realizar a tradução e adaptação cultural do *Behavior Change Protocol* para as práticas educativas em *Diabetes Mellitus*.

**Método:**

estudo metodológico de adaptação cultural, no qual foram executadas as etapas de tradução, retrotradução, avaliação por comitê de juízes e pré-teste do instrumento aplicado a uma amostra de 30 usuários com *diabetes mellitus* tipo 2.

**Resultados:**

a avaliação do instrumento pautou-se pelos critérios de equivalência semântica, idiomática, conceitual e cultural entre o instrumento original e o traduzido, sendo que o Índice de Validade de Conteúdo médio apresentado foi de 0,85.

**Conclusão:**

o instrumento apresentou validade de conteúdo indicativa de boa adaptação à cultura brasileira para seu uso em práticas educativas orientadas para o autocuidado em *diabetes mellitus* tipo 2.

## Abstract (es)

**Objetivo:**

realizar la traducción y adaptación cultural del instrumento *Behavior Change Protocol* para las prácticas educativas en Diabetes Mellitus.

**Método:**

estudio metodológico de adaptación cultural, comprendiendo las etapas de traducción, retrotraducción, evaluación por un comité de jueces y pretest del instrumento, en una muestra de 30 usuarios del servicio de salud pública con Diabetes Mellitus tipo 2.

**Resultados:**

la evaluación del instrumento se pautó en los criterios de equivalencia semántica, idiomática, conceptual y cultural entre el instrumento original y el traducido, habiendo presentado Índice de Validez de Contenido promedio de 0,85.

**Conclusión:**

el instrumento presentó validez de contenido indicativa de adaptación satisfactoria a la cultura brasileña para ser utilizado en prácticas educativas dirigidas al autocuidado en DM tipo 2.

## Introduction

In recent years, educational interventions in diabetes mellitus (henceforth DM) have been redesigned to consider healthcare service users as protagonists in the construction of their self-care. This approach requires the development of abilities that enable healthcare professionals to establish links with DM healthcare service users, providing qualified assistance centered around their needs^1-3^.

Education in DM service users self-care is deemed essential for increasing their level of knowledge about their chronic condition and prevention of its consequences, as well as for strengthening their motivation to follow a suitable therapeutic plan. Furthermore, education may contribute to users overcoming barriers related to social and emotional factors that may affect their quality and length of life^4-5^.

Bearing upon this approach, specialists from the University of Michigan (USA) developed the Behavior Change Protocol, an instrument aimed at assisting healthcare professionals in applying educational practices that stimulate DM healthcare service users empowerment. Empowerment in healthcare is a skill-developing process whereby healthcare service users acquire self-control to deal with their chronic condition and make decisions aligned with healthy lifestyle habits^6-7^.

Studies^1-4^ have been using the Behavior Change Protocol in educational programs on DM, the results of which have been favorable in terms of change of attitude and behavior and have led to improvements in glycemic control by participating healthcare service users.

A literature review^8^ revealed lack of instruments adequate to be used in educational practices for addressing Brazilian healthcare service users’ behavioral, psychosocial and clinical aspects, aiming at self-care promotion. In this regard, the Behavior Change Protocol stands out as an instrument that values subjective aspects of care and stimulates DM healthcare service users to manage their health condition through educational practices that lead to reflection, joint responsibility, and informed decision-making in self-care behavior^1-3,6^.

Given the need for an adequate instrument to promote Brazilian DM healthcare service users’ empowerment drawing on self-care practices, a group of researchers at the Nursing School, together with researchers at the Laboratory for Experimentation in Translation and the Statistics Department at the Federal University of Minas Gerais, decided to carry out the present study herein reported under the auspices of the project Empodera [Empower - methodological innovation in educational practices aimed at autonomy in healthcare].

The aim of our study was to translate and cross-culturally adapt the Behavior Change Protocol for educational practices in DM.

## Method

This is a methodological study, carried out from June 2015 to January 2016, which pursued the translation and cross-cultural adaptation of a healthcare instrument following the steps adopted in the literature, namely 1) forward translation; 2) synthesis; 3) back-translation; 4) expert committee assessment and 5) pre-test^9-11^.

The Behavior Change Protocol is made up of 25 questions and an appendix of 11 questions tiltled “I-SMART”. The 25 unstructured questions are open questions grouped into five domains: Problematization (1- problem definition); Feelings (2- recognition of feelings); Goals (3- goal choosing); Systematization of care (4- development of a care plan to reach goal(s) - My Intelligent Plan); Assessment (5- healthcare service user experience and evaluation of care plan)^4-6^. The protocol aims to assist healthcare professionals to develop educational practices in Diabetes Mellitus with the aim of leading users to conceive of and develop a care plan that fosters a change in their behavior. It is a guide to emancipatory educational practice, whereby users have a leading role in the planning, decision-making and execution of healthcare actions, while at the same time being able to identify their problems and challenges, define needs, understand limitations and promote adequate actions to deal with daily situations.

Two Brazilian translators, proficient in English and with a degree in translation, carried a forward translation of the Behavior Change Protocol, yielding versions T-1 and T-2. The two versions were then synthesized into version T1-2 and submitted to assessment by a third translator, who holds a PhD in applied linguistics. The suggested changes were analyzed by the forward text translators and adopted, when relevant, in a synthesis-version denominated T-12.

Two additional Brazilian translators, also proficient in English and with a translation degree, carried out back-translations. Subsequently, these versions were compared and used to identify possible divergences that could reveal the need to review some of the renditions. Conflicts regarding the original text were discussed and solved, and necessary changes were introduced in version T-12, yielding version T-13, which was then submitted to assessment by the expert committee.

Assessment was carried out in a face-to-face meeting by an expert committee, which was made up of an interdisciplinary team of eight healthcare professionals and applied linguists. Inclusion criteria for committee members were: holding a degree in healthcare sciences or applied linguistics, having experience in DM care or having taken part in research on translation and cross-cultural adaptation. The assessment was based on analysis of semantic, idiomatic, conceptual and cultural equivalence.

Each committee member received an invitation letter introducing the study and requesting them to assess the translated content regarding adequacy and acceptability. The assessment consisted in assigning each item in the protocol one of the three possible scores: 1) item needs to be re-translated on the whole; 2) item needs to be re-translated in part; 3) item does not need to be re-translated.

Experts worked individually on comparing the original English version to version T-13 for an initial 90-minute period, followed by a round-table discussion on adequancy and acceptability of each translated item. Any disagreement on a word or term was discussed until consensus was reached about the most accurate rendition and the one that construed meaning analogous to that construed in the original instrument.

The Content Validity Index (CVI) defined by the sum of the relative frequencies of responses with scores 2 and 3 , was used to verify the level of agreement of the experts regarding adequacy of the assessed items. A CVI greater than or equal to 0.78 was considered to indicate correspondence with the original text, both for each item and for the global instrument^11^.

Edited changes, as well as arguments accounting for them, were tape-recorded and transcribed by the researchers. The outcome of this stage was a first consensual, equivalent version of the Protocol in Brazilian Portuguese (version T-14), named “*Protocolo Mudança de Comportamento em Diabetes Mellitus*” (Behavior Change Protocol in Diabetes Mellitus).

The translated version was used in pre-testing, administered through individual face-to-face interviews with thirty healthcare service users with a type 2 DM diagnosis. Inclusion criteria were: participants aged between 30 and 75, of either sex, capable to listen to and verbally respond to the questions in the instrument, and not having complications related to DM (neuropathy, nephropathy, retinopathy and cardiopathy, among others), since the protocol is meant to be used in programs to prevent chronic complications.

For interviews, home visits were arranged in advance by telephone call with healthcare service users who agreed to participate in the study. Interviews were carried out by a responsible for instrument administration together with a nursing student so as to record observations. The aim was to evaluate the clarity of the questions, identify problems related to the users’ understanding of the questions, and identify difficulties found by those responsible for administrating the instrument. Instrument administration time ranged from 20 to 40 minutes.

A form was used to record the level of healthcare service user’s understanding of the questions with the following options: 1) User had no difficulty in understanding the question; 2) User had some difficulty in understanding the question; 3) User requested the question to be repeated more than once; and 4) User did not reply to the question. Audio recordings were made during the meetings, along with comments and suggestions by the instrument administrators.

Interpretation difficulties related to the questions or specific vocabulary of the protocol were treated as potential problems and were solved from an interdisciplinary perspective. Specialists from the expert committee and the instrument administrators took part in this step. Adjustments were made and questions were posed to the target public to test solutions until all problems were deemed to be solved. A final version of the Behavior Change Protocol in Diabetes Mellitus was thus obtained, labelled T-15, a cross-culturally adapted version to spoken Brazil Portuguese.

The study’s approval is found in *Plataforma Brasil* under decision No. CAAE 41225015.0.0000.5149. The participants signed an Informed Consent Form in accordance with Resolution 466/12 of the National Health Council.

## Results

The Behavior Change Protocol was translated and cross-culturally adapted into Brazilian Portuguese and named “*Protocolo Mudança de Comportamento em Diabetes Mellitus*” (Behavior Change Protocol in Diabetes Mellitus). Translation and cross-cultural adaptation of the instrument followed the methodology set up in the literature. Changes made to the translated items were based on the suggestions by specialists, researchers, and healthcare service users, with the aim of improving clarity and understanding by the target population.

The two texts obtained by forward translation, T-1 and T-2, achieved similar results; thus, only a few adjustments were deemed necessary by the translators to obtain a synthesis version - T-12. This version was then back-translated by two independent translators. The two back-translated versions were analyzed and, based on this analysis, necessary adjustments were made to the synthesis version, yielding an updated version labelled T13. This version was found to accurately construe the meaning in the instrument that was submitted to expert assessment.

The expert committee was made up by three nurses, a dietician, a physiotherapist and three applied linguists. 62.5% of experts had a master’s or doctorate degree in healthcare education or translation, which points to the relevance of the academic background of the expert committee for contributing with a cross-culturally adaptation study. All the experts reported being proficient in reading comprehension in English.

Most of the suggestions involved rewriting, such as word order in a clause or replacement of a given term by a synonym. Table 1 shows the CVI calculated for each question in the protocol after experts’ assessment of T-13. The higher the CVI value, the lower the number of changes deemed necessary to improve the renditions. Eleven (44.0%) out of the twenty-five items presented a score below 0.78 and were discussed by the committee until reaching consensus. Mean CVI of the protocol was 0.85 (standard deviation=0.1).

**Table 1:** Content Validity Index for each question in the Behavior Change Protocol in Diabetes Mellitus according to experts’ assessment. Belo Horizonte, MG, Brazil, 2016

Experts’ score																	
Question	1		2		3		4		5		6		7		8		CVI*
1	3		2		3		3		2		3		3		3		1.00
2	3		1		3		2		3		1		3		3		0.75
3	3		3		3		2		3		3		2		3		1.00
4	1		3		3		3		3		3		1		3		0.75
5	3		1		1		3		1		1		3		1		0.38
6	3		3		3		3		3		3		1		3		0.88
7	1		1		3		2		3		3		1		3		0.63
8	3		1		3		1		3		3		3		3		0.75
9	3		3		2		2		2		2		3		2		1.00
10	3		1		3		3		3		1		3		3		0.75
11	3		2		3		2		3		3		3		3		1.00
12	1		3		3		3		3		3		1		3		0.75
13	3		3		2		3		3		3		3		3		1.00
14	3		3		3		2		3		3		3		3		1.00
15	3		3		2		3		3		3		3		3		1.00
16	2		3		3		1		3		2		1		3		0.75
17	3		3		2		3		3		1		2		3		0.88
18	3		2		3		3		3		3		2		2		1.00
19	3		3		3		3		3		1		1		1		0.63
20	3		3		3		3		3		3		1		1		0.75
21	1		2		2		1		3		3		3		3		0.75
22	3		3		3		3		3		3		3		1		0.88
23	3		2		3		3		3		3		3		3		1.00
24	3		3		3		3		3		3		3		3		1.00
25	1		2		2		2		3		3		3		3		0.88
Mean CVI*																	0.85

*CVI – Content Validity Index

The CVI for the appendix “My Intelligent Plan” was calculated after experts’ assessment of version T-13. Three (27.3%) out of the eleven questions presented a score below 0.78. Mean Content Validity Index of the appendix was 0.85 (standard deviation= 0.1) and is shown in Table 2.

**Table 2:** Content Validity Index for each question in the “My Intelligent Plan” appendix according to experts’ assessment. Belo Horizonte, MG, Brazil, 2016

Experts’ Score									
Question	1	2	3	4	5	6	7	8	CVI*
1	3	3	3	1	3	3	3	3	0.88
2	3	3	3	3	3	2	3	2	1.00
3	2	3	2	3	3	3	3	3	1.00
4	3	3	1	3	3	2	1	1	0.63
5	3	3	3	3	3	3	1	1	0.75
6	3	3	3	3	3	3	3	1	0.88
7	3	2	2	3	3	3	3	1	0.88
8	3	3	3	3	3	3	3	3	1.00
9	3	1	3	1	3	2	1	1	0.50
10	3	3	3	2	3	3	3	1	0.88
11	3	3	3	3	3	3	3	2	1.00
Mean CVI*									0.85

*CVI – Content Validity Index

A total of 30 healthcare service users with type 2 DM were interviewed during the pre-test step, all of which were Brazilian citizens residing in Belo Horizonte-MG. Users were mostly female (70%), 60 years old or older (74%), with a monthly income of two minimum salaries (43.3%) and five years or more since DM diagnosis (55.6%).

The results of this step revealed that, despite the modifications to the renditions suggested by the experts, some questions in the instrument were not easily understood, as some words and expressions proved confusing and ambiguous. Three administrations of the Behavior Change Protocol in Diabetes Mellitus and two interdisciplinary meetings were necessary until problems with instrument comprehension by the target public were no longer detected.

The study considered null frequency of comprehension problems an indicator of full understanding of the instrument by target users. “My Intelligent Plan” was administered only once, no comprehension problems found on the part of the target public. Table 3 shows difficulties found by administrators and interviewees in asking and replying to the questions at each protocol administration.

**Table 3:** Percentage of interviewed individuals presenting comprehension difficulties and of administrators showing difficulty in protocol administration according to each question in the instrument and its version. Belo Horizonte, MG, Brazil, 2016

Question	Administration 1		Administration 2		Administration 3	
	Interviewee (n*=9)	Administrator (n*=9)	Interviewee (n*=14)	Administrator (n*=14)	Interviewee (n*=7)	Administrator (n*=7)
1	3 (33%)	0 (0%)	1 (7%)	1 (7%)	0 (0%)	0 (0%)
2	4 (44%)	0 (0%)	1 (7%)	1 (7%)	0 (0%)	0 (0%)
3	3 (33%)	1 (11%)	1 (7%)	3 (21%)	0 (0%)	1 (14%)
4	3 (33%)	5 (55%)	3 (21%)	3 (21%)	0 (0%)	0 (0%)
5	4 (44%)	0 (0%)	1 (7%)	1 (7%)	0 (0%)	0 (0%)
6	3 (33%)	0 (0%)	2 (14%)	2 (14%)	0 (0%)	2 (28%)
7	4 (44%)	0 (0%)	2 (14%)	3 (21%)	0 (0%)	0 (0%)
8	4 (44%)	3 (33%)	1 (7%)	0 (0%)	0 (0%)	0 (0%)
9	4 (44%)	2 (22%)	7 (50%)	7 (50%)	0 (0%)	0 (0%)
10	3 (33%)	0 (0%)	1 (7%)	3 (21%)	0 (0%)	0 (0%)
11	3 (33%)	3 (33%)	0 (0%)	0 (0%)	0 (0%)	0 (0%)
12	0 (0%)	0 (0%)	0 (0%)	0 (0%)	0 (0%)	0 (0%)
13	4 (44%)	1 (11%)	2 (14%)	2 (14%)	0 (0%)	0 (0%)
14	3 (33%)	1 (11%)	5 (35%)	2 (14%)	0 (0%)	1 (14%)
15	5 (55%)	2 (22%)	3 (21%)	1 (7%)	0 (0%)	0 (0%)
16	4 (44%)	2 (22%)	2 (14%)	1 (7%)	0 (0%)	0 (0%)
17	3 (33%)	2 (22%)	3 (21%)	1 (7%)	0 (0%)	0 (0%)
18	3 (33%)	2 (22%)	2 (14%)	1 (7%)	0 (0%)	1 (14%)
19	0 (0%)	0 (0%)	0 (0%)	0 (0%)	0 (0%)	0 (0%)
20	4 (44%)	2 (22%)	2 (14%)	1 (7%)	0 (0%)	0 (0%)
21	4 (44%)	1 (11%)	5 (35%)	2 (14%)	0 (0%)	0 (0%)
22	0 (0%)	1 (11%)	2 (14%)	1 (7%)	0 (0%)	0 (0%)
23	4 (44%)	1 (11%)	1 (7%)	1 (7%)	0 (0%)	0 (0%)
24	0 (0%)	1 (11%)	2 (14%)	3 (21%)	0 (0%)	1 (14%)
25	3 (33%)	1 (11%)	2 (14%)	2 (14%)	0 (0%)	1 (14%)

*n- number of individuals interviewed

Experts’ comments deemed relevant to translation and cross-cultural adaptation were had to do with: subtitles for the different sections in the instrument, adjustments in conceptual meaning, explanation of meaning for greater clarity of comprehension in the context of Brazilian culture, and misinterpretation or difficulty in comprehension by the target public.

The subtitles for the different sections in the instrument were verbs: “define” “recognize”, “choose”, “make”, and “experience & evaluate”. These verbs name the five steps in the protocol and do not actually operate as commands to carry out an action. These terms were translated as nouns in the final version T-15: “*definição*” (definition), “*identificação*” (identification), “*definição*” (definition), “*elaboração*” (preparation) and “*avaliação e experiência*” (evaluation and experience).

Conceptual adjustments were also necessary in question 21, since experts anticipated that short answers like “good” or “bad” and/or “yes” or “no” could be expected. Thus, the question “*Seguir o plano foi bom ou ruim? Ele ajudou você a controlar o diabetes?*” (“Was following the plan good or bad? Did it help you control your diabetes?”) was rephrased as “*Como foi seguir o plano?*” (“What was it like to follow the plan?”) in versions T-14 and T-15. The objective of the question in the English version is for the user to reflect and report on how it has been to follow the plan and pursue its goals, seeking to identify barriers to and facilitators of self-care.

In questions 2, 4, 5, 10, 11 and 16, meaning explicitation was necessary in order to improve comprehension by Brazilian interviewees. In question 4, the English word “thoughts” had two possible renditions in Brazilian Portuguese: “*pensamento*” (idea) or “*opinião*” (opinion). The translated question “*como é que você se sente tendo o diabetes?*” (“How do you feel about having diabetes?”) in version T-13 was rephrased as “*O que você acha de ter diabetes?*” (“What are your thoughts about having diabetes?”) in version T-14. However, in the pre-test phase, this question still raised comprehension problems and had to be rephrased as “*Como você se sente com essa situação de ter de cuidar da sua saúde?*” (“How do you feel about this situation of having to take care of your health?”) in version T-15.

In question 5, experts considered it important to explain what the commands “insert feeling” and “insert meaning” meant in the original version. These commands had been rendered as “*preenchido pelo aplicador*” (“to be filled in by administrator”) in version T-13 and were then rephrased as “*insira o(s) sentimento(s) identificado(s) pelo usuário*” (“insert the feeling(s) mentioned by user”) and “*insira o(s) significado(s) desse(s) sentimento(s) para vida do usuário*” (“insert the impact of those feelings on user’s life”) in version T-14. However, in the pre-test phase, the administrators did not feel confident in carrying out this line of questioning, since the structure of the question was long and not very clear; hence, the question was rephrased as “*Você se sente assim (inserir os sentimentos expostos pelo usuário) por quê?*” (“Why do you feel (insert feelings mentioned by user)?”) in version T-15.

In question 11, as a result of ambiguity problems during pre-testing, the question “*Tem alguma pessoa que possa ajudá-lo?*” (“Is there anybody that can help you?”) in version T-14 had to be rephrased as “*Tem alguma pessoa que possa ajudá-lo a conquistar as suas metas*” (“Is there anybody that can help you achieve your goals?”) in version T-15.

During pre-testing, questions 12, 16 and 17 were not easily understood by interviewees. In question 12, the words “*vantagens*” (“advantages”) and “*desvantagens*” (“disadvantages”) caused comprehension problems, since many users claimed not to understand their meaning. Therefore, the question “*Pense nas escolhas que você faz para a sua saúde. Quais as vantagens e desvantagens de cada uma delas*” (“Think about the choices you make for your healthcare. What are the advantages and disadvantages of each choice?”) was rephrased as “*Pense nas escolhas que você faz para a sua saúde. Qual o lado bom e o lado ruim de cada uma delas?*” (“Think about the choices you make for your healthcare. What is good and bad about each choice?”)

Renditions for questions 16 and 17 required rephrasing, as they requested the user to give a score ( quantitative data). The strategy was to use a qualitative scale instead of a numerical scale. For example, the command “*Dê uma nota de 1 a 10 para a importância de superar as dificuldades relacionadas à sua saúde*” (“Give a score from 1 to 10 to how important it is for you to overcome your health problems”) in version T-14 was rephrased in the pre-test phase so that the interviewee, rather than a giving a score, could choose one options within a scale in decreasing order of importance: “a) É *muito importante superar as dificuldades relacionadas à sua saúde* (It is very important to overcome your health problems); b) É *importante superar as dificuldades relacionadas à sua saúde* (It is important to overcome your health problems); c) É “*mais ou menos” importante superar as dificuldades relacionadas à sua saúde* (It is “more or less” important to overcome your health problems); d) *Não é importante superar as dificuldades relacionadas à sua saúde* (It is not at all important to overcome your health problems).”

Analogously, the command “*Dê uma nota de 1 a 10 para a sua confiança em alcançar a sua meta*” (Give a score from 1 to 10 to rate your confidence in achieving your goal) was rephrased as a question “*Como você vê sua confiança para alcançar a(s) sua(s) meta(s)”* (How confident do you feel in achieving your goal(s)). The scale options were : a) *Sinto-me muito confiante* (I feel very confident); b) *Sinto-me confiante* (I feel confident); c) *Sinto-me mais ou menos confiante* (I feel kind of confident); d) *Não me sinto confiante* (I don’t feel confident at all)”.

These examples demonstrate the importance of testing and adapting the questionnaire by working with interviewees during the pre-test phase. Insights gathered from interviews can enhance and bring improvements in addition to those brought by the suggestions made by the expert committee. In our study, experts had not pointed out any problem whatsoever regarding score ratings and numerical scales, this problem having arisen when the translate dinstrument was administered to a sample of healthcare service users emulating actual administration of the protocol.

The version obtained after discussing and introducing rephrases drawing upon interviewees’ feedback was labelled T-15 and became the final version of the Behavior Change Protocol in Diabetes Mellitus cross-culturally adapted to spoken Brazilian Portuguese, available for download at the Empodera project website^1^*.

## Discussion

The study herein reportedaimed at translating and cross-culturally adapting the Behavior Change Protocol for educational practices in DM in Brazil.

The Brazilian version of the Behavior Change Protocol, named *Protocolo Mudança de Comportamento em Diabetes Mellitus,* was considered adequate and acceptable by experts in healthcare and applied linguistics. The strategy of using an interdisciplinary expert committee favored problem recognition and solution in the translated version, obtaining semantic, idiomatic, conceptual and cultural equivalence between original and translated items and enhancing data analysis in the pre-test phase^9^.

The pre-test phase was conducted through face-to-face interviews that aimed to get feedback on the translated version at work in a setting emulating actual protocol administration. Language discussions drew on the Systemic Functional Theory, which enabled critical analysis of the text to be cross-culturally adapted, by considering aspects of written and spoken language, as well as other intervening semiotic systems such as interviewees’ body language, gestures and gaze^11-15^.

As such, the instrument was cross-culturally adapted to be used as spoken text, aiming to facilitate interaction and user understanding within a typical setting in Brazilian cultural context. In our study, null frequency of comprehension problems was sought as an indicator of optimal comprehension by interviewees. This differs from previous studies, which use a 15% of higher frequency to review renditions that cause comprehension problems in the pre-test phase^16^. Null frequency was a major criterion for the authors to consider the final version, labelled T-15, cross-culturally adequate and acceptable to be used with a Brazilian target population.

The target population’s profile proved to be a determining factor for the rephrases carried out. The target population consisted of healthcare service users with varied levels of literacy, a fact that needs to taken into account to achieve accessible language, as recommended the literature^10,17^.

It is important to highlight that conducting educational practices guided by this instrument requires the healthcare professional to develop skills in dealing with healthcare service users with DM. These skills are developed through a continuous process of training and reflection upon the practices daily carried out. The developed skills can be expressed by behaviors such as knowing how to listen to users, accepting different opinions, having empathy and the capacity to work together with the healthcare service user to build knowledge^18^.

Items making up the Behavior Change Protocol in DM are consistent with major elements in DM approaches as revealed in the findings of previous studies in which healthcare service users reported that barriers to the practice of self-care are related to psychosocial, behavioral and economic factors. These barriers may explain why a considerable number of healthcare service users do not manage to follow a dietary plan, carry out physical activities or adhere to drug treatment, the extrinsic support and motivation of the healthcare professional being essential to healthcare planning and advising^18-20^.

The instrument aims to promote healthcare service user reflection and problematization of their daily life, exploring barriers and feelings involved in daily care. Agreement between healthcare professional and users on the preparation of a plan and goals is considered a prerequisite to obtaining good results regarding glycemic control and treatment satisfaction^6^.

Our study relied on a methodology for translation and cross-cultural adaptation of healthcare instruments described in the literature^21^, with a particular concern about having an interdisciplinary expert committee, which enhanced the cross-cultural adaptation process informed by valuable insights provided by its members based on their areas of expertise. Furthermore, the pre-test dynamics followed in our study allowed cross-cultural adaptation through careful analysis of results gathered after each subsequent round of face-to-face interviews until comprehension problems were fully solved out.

This study contributes to nursing practice regarding planning and systematization of educational practices focusing on DM through the the adoption of the Behavior Change Protocol in DM as a guide to assist the healthcare service user in their self-care practices towards empowerment.

## Conclusion

The translated and cross-culturally adapted instrument showed content validity indicating adequacy and acceptability to be used in educational practices in type 2 DM aimed at building healthcare service user empowerment.
